# Alcohol spectrum disorders in women : a review of current data

**DOI:** 10.1192/j.eurpsy.2023.125

**Published:** 2023-07-19

**Authors:** F. Thibaut

**Affiliations:** Dept of Psychiatry and Addiction, University Paris Cité - University Hospital Cochin (APHP), Paris, France

## Abstract

**Abstract:**

Epidemiological studies indicate an alarming narrowing in the gender gap of alcohol and tobacco use especially in adolescents, which may reflect changes in sociocultural patterns in women. Moreover, women use more often alcohol to cope with stress and negative feelings. Pharmacokinetics and pharmacodynamics differences, reward process specificities, and female hormones play a major role in gender differences. Women’s consumption of alcohol may be associated with serious birth and developmental consequences in newborns.Thibaut F et al. WFSBP and IAWMH Guidelines for the treatment of alcohol use disorders in pregnant women. The World Journal of Biological Psychiatry 2019 20(1):17-50

**Disclosure of Interest:**

None Declared

